# Can ROMA algorithm stratify ovarian tumor patients better when being based on specific age ranges instead of the premenopausal and postmenopausal status?

**DOI:** 10.1007/s13277-015-4733-z

**Published:** 2016-01-11

**Authors:** Anita Chudecka-Głaz, Aneta Cymbaluk-Płoska, Jolanta Jastrzębska, Janusz Menkiszak

**Affiliations:** 1 Department of Gynecological Surgery and Gynecological Oncology of Adults and Adolescents, Pomeranian Medical University, Al. Powstańców Wielkopolskich 72, 70-111 Szczecin, Poland; 2 High School Number 2 in Szczecin, Szczecin, Poland

**Keywords:** HE4, CA125, ROMA algorithm, Ovarian cancer, Diagnosis

## Abstract

After several years of research, HE4 was found to be characterized by slightly worse sensitivity but significantly higher specificity as compared with CA125. Further studies led to the diagnostic potential of both markers (CA125 and HE4) being combined in a single risk of malignancy algorithm (ROMA) algorithm. The objective of this study was to assess the diagnostic capabilities of the ROMA algorithm using age ranges instead of dichotomization of patients according to the pre- and postmenopausal status. A total of 413 female patients were included in the study, including 162 premenopausal and 251 postmenopausal women. Calculation of the final ROMA values was achieved by means of stepwise reduction of coefficients in the proposed formula of: %ROMA = exp(PI)/[1-exp(PI)]*100) and PI = A + W(HE4)^*^ln(HE4) + W(CA125)^*^ln (CA125) and the arrangement of values with consideration to the age group, HE4 level, differentiation of modification, and directional coefficients as well as determination of individual deviations affecting the widening of the median. The cutoff value of modified algorithm ROMA P for the entire study population was calculated from receiver operating characteristic (ROC) curve and DeLong method at the levels of 23.5 %. Marked higher sensitivity and negative predictive value (NPV) values are observed for the standard ROMA algorithm while higher specificity and positive predictive value (PPV) values are observed for the modified algorithm ROMA P. The proposed age-related modification of algorithm calculation does not require the patients being dichotomized according to their pre- or postmenopausal status, and satisfactory diagnostic values may be obtained using a single cutoff point for the entire population.

## Introduction

Ovarian cancer is responsible for the largest number of deaths due to gynecological cancers in European and North American women. Worldwide, it is second only to cervical cancer. Annual incidence of ovarian cancer is estimated at more than 200,000 cases while the mortality is estimated at over 150,000 of deaths [1]. Currently, we have no sufficient capabilities to perform ovarian cancer screening examinations in the entire population of women. The partial screening program includes high-risk patients with BRCA 1 or BRCA 2 gene mutations [2]. Therefore, the main stress is placed on the possibilities of earlier diagnostics and identification of ovarian cancer at earlier stages of the disease. Clinical staging remains one of the most important prognostic factors that impact the survival parameters in patients [3]. The first marker used in routine diagnostics of ovarian cancer was the antigen CA125, widely used to date [4]. In 2003, Hellstrom et al. [5] were the first to report the potential use of HE4 as a marker in the diagnostics of ovarian cancer. After several years of research, HE4 was found to be characterized by slightly worse sensitivity but significantly higher specificity as compared with CA125 [6–11]. Further studies led to the diagnostic potential of both markers (CA125 and HE4) being combined in a single risk of malignancy algorithm (ROMA) [12–16]. According to numerous studies conducted to date, ROMA appears to be one of the best methods for stratification of ovarian tumor patients into groups of high vs. low risk of ovarian cancer. The algorithm is objective, easy to perform, inexpensive, and characterized by appropriate sensitivity and specificity [12, 13]. However, the search for methods for achieving better results in terms of the algorithm’s sensitivity and specificity is ongoing [17, 18].

The objective of this study was to assess the diagnostic capabilities of the ROMA algorithm using age ranges instead of dichotomization of patients according to the pre- and postmenopausal status.

## Materials and methods

### Patients

A total of 413 female patients were included in the study, including 162 premenopausal and 251 postmenopausal women. Patients were diagnosed and treated at the Department of Gynecological Surgery and Gynecological Oncology Adults and Adolescents in Szczecin, Poland, between 2011 and 2015. Qualified for the study were the consecutive patients who attend the hospital presenting with ovarian tumor, ovarian cyst, or ascites (suspected ovarian cancer). After informed consent was obtained, blood samples were collected simultaneously and serum levels of HE4 and CA125 were determined on current basis without freezing at the hospital’s Central Laboratory. All the samples were taken before diagnosis, not in the follow-up period. Both markers are routinely determined in all patients reporting to our clinic with adnexal lesions. ROMA algorithm analyses based on specific age groups were performed in a retrospective manner after an appropriate formula was determined. In the clinical decision-making process, each marker was analyzed separately. After histopathological examination results were obtained, patients were finally qualified into one of the two groups:

A. Patients with ovarian cancer
B. Patients with benign adnexal lesions

Patients with history of chronic renal diseases, history of chronic lung diseases, metastatic ovarian tumors, ongoing treatment of other cancers, or presenting with elevated creatinine levels were not qualified to the study. A detailed division of patients in both groups is presented in Table 1.

**Table 1:** Patients characteristics

	Ovarian cancers *n* (%)	Benign diseases *n* (%)
	162	251
Age		
Median	59.7 (24–90)	35 (18–88)
Range		
Hormonal status		
Premenopausal	38 (23.5)	214 (85.3)
Postmenopausal	124 (76.5)	37 (14.7)
Age interval		
W1 ( <20 YO)	–	38 (15.1)
W2 (21–30)	3 (1.9)	55 (21.9)
W3 (31–40)	7 (4.3)	73 (29.1)
W4 (41–50)	28 (17.3)	48 (19.1)
W5 (51–60)	47 (29)	22 (8.8)
W6 (61–70)	43 (26.5)	10 (4)
W7 (71–80)	25 (15.4)	4 (1.6)
W8 (>81)	9 (5.6)	1 (0.4)
Ovarian cancer histopathology		NA
Serous	132 (81.5)	NA
Mucinous	9 (5.6)	NA
Clear cell	8 (4.9)	NA
Endometrioid	13 (8)	NA
Oarian cancer FIGO stage		NA
Stages I and II	54 (33.3)	NA
Stages III and IV	105 (64.7)	NA
Ovarian cancer grade		NA
Grade 1	34 (21)	NA
Grade 2	54 (33.3)	NA
Grade 3	74 (45.7)	NA
Benign tumor histopathology		
Endometriosis	NA	120 (47.8)
Teratoma	NA	43 (17.1)
Follicular cysts	NA	33 (13.1)
Paraovarian cysts	NA	26 (10.4)
Hemorrhagic cysts	NA	28 (11.6)

Median values and ranges were determined for both groups and all respective subgroups, with appropriate comparisons being made with respect to the levels of HE4 and CA125, ROMA algorithm calculated in the standard manner (ROMA) as well as the modified ROMA algorithm (ROMA P). Sensitivity, specificity, as well as positive and negative predictive values of CA125 and HE4 were determined for the standard and the modified algorithm. Additionally, diagnostic usefulness of each marker was determined using ROC-AUC.

Calculation of the final ROMA values was achieved by means of stepwise reduction of coefficients in the proposed formula of:

- %ROMA = exp(PI)/[1-exp(PI)]*100,
- PI = *A* + *W*(HE4)^*^ln(HE4) + *W*(CA125)^*^ln (CA125),
- Below 20 years old: *A* = −12; *W*(HE4) = 2.38; *W*(CA125) = 0.063,
- From 21 to 30 years old: *A* = −11.44; *W*(HE4) = 2.19; *W*(CA125) = 0.158,
- From 31 to 40 years old: *A* = −10.88; *W*(HE4) = 2; *W*(CA125) = 0.254,
- From 41 to 50 years old: *A* = −10.32; *W*(HE4) = 1.81; *W*(CA125) = 0.349,
- From 51 to 60 years old: *A* = −9.77; *W*(HE4) = 1.61; *W*(CA125) = 0.445,
- From 61 to 70 years old: *A* = −9.21; *W*(HE4) = 1.42; *W*(CA125) = 0.541,
- From 71 to 80 years old: *A* = −8.65; *W*(HE4) = 1.23; *W*(CA125) = 0.636,
- Above 80 years old: *A* = −8.09; *W*(HE4) = 1.04; *W*(CA125) = 0.732.

In the calculation, we used also the arrangement of values with consideration to the age group, HE4 level, differentiation of modification and directional coefficients, as well as determination of individual deviations affecting the widening of the median.

### Laboratory methods

The HE4 serum levels of the marker were determined using the Roche Elecsys^®^ assay on a Cobas e601 apparatus. This is a one-step sandwich electro-chemiluminescence immunoassay (ECLIA) for quantitative determination of human epididymal protein 4. The detection range for HE4 was 15–1500 pmol/l; in case of values exceeding 1500 pmol/l, the samples were diluted in a 1:20 ratio using Elecsys Diluent.

The CA125 serum levels of the marker were determined using the ARCHITECT CA125 II assay on an ARCHITECT 2200SR System. This is a two-step immunoassay to determine the presence of CA125 antigen using Chemiluminescent Microparticle Immunoassay (CMIA) technology.

CA125 and HE4 assays were carried out according to manufacturers’ instructions, with appropriate controls testing within the normal ranges. The detection range for CA125 was 1–35 U/ml.

### Statistical analysis

Descriptive characteristics of the examined population of patients were prepared, including the minimum, maximum, mean, and median values. Also, the scatter diagrams of the empirical values of markers were plotted for individual study groups. The mean/median values in individual groups and subgroups were compared using the nonparametric Mann–Whitney’s *U* test.

The contingency table was used in the assessment of diagnostic usefulness of CA125 and HE4 assays and ROMA values and subsequent calculation of the following parameters:

- Sensitivity = TP/TP + FN
- Specificity = TN/FP + TN
- Positive predictive value (PPV) = TP/TP + FP
- Negative predictive value (NPV) = TN/FN + TN

The diagnostic performance was studied using receiver operating characteristic (ROC) curves based on continuous variables. HE4, CA125, and ROMA represented diagnostic variables acting as stimulants which increase the probability of ovarian cancer proportionally to their rising value. The area under curve (AUC), standard error (SE_AUC_), and confidence interval (CI_AUC_) values for AUC were calculated according to the nonparametric method of DeLong. We used this method to compare AUCs considering the fact that measurements of HE4, CA125, and ROMA were done for the same objects (groups of patients). The level of significance was taken as *p* < 0.05.

## Results

### Patient, biomarker, and algorithm baseline characteristics

Detailed characteristics of patients are presented in Table 1. The analysis included a total of 162 patients with ovarian cancer. As much as 81.5 % of these cancers were serous and 64.7 % of serous was of a high clinical stage. The group of benign gynecological disorders consisted of 251 patients, 47.8 % of those being diagnosed with endometriosis. Also presented in Table 1 is the distribution of patients into individual age groups used for modification of the ROMA algorithm.

Medians and ranges or marker levels as well as values obtained using the standard and the modified ROMA algorithm are presented in Table 2. Comparative analysis between the values from the standard and the modified algorithm revealed no statistically significant differences within the analyzed groups and subgroups. The only clearly evident difference was related to benign disorders being pooled regardless of histopathological type, where the values obtained using the modified algorithm were significantly higher (6.7 vs. 8.29 %, *p* = 0.0001).

**Table 2:** Serum CA125, HE4, ROMA, and ROMA P levels according to age, histology, FIGO stage, and tumor grade

	CA125 (U/ml)		HE4 (pmol/l)		ROMA (%)		ROMA P (%)		p (ROMA vs. ROMA P)
	Median	Range	Median	Range	Median	Range	Median	Range	
Ovarian cancer (all)	397.5	9–7459.1	340.7	12–9264	90.3	0.5–100	91.46	1.33–99.9	0.8641
Serous	421.1	9–7459.1	390.7	15–9264	91.9	0.5–100	92.5	1.3–99.9	0.7963
Mucinous	58.8	11.3–600	69.2	15–538	30.1	6.2–95.8	27.2	1.9–96.1	0.9699
Clear cell	389.9	95.8–1725.5	184.8	49.5–849.9	81.5	8.1–98.2	73.7	15.6–98.3	0.9581
Endometrioid	00	41.5–2996.8	340.7	46.1–1235	94.6	21–99.3	93.2	2.5–99.4	0.9591
FIGO stages I and II	130.3	9–2347	79	15–1235	35.3	0.5–99.3	33.6	1.3–99.3	0.8610
FIGO stages III and IV	591	18–7459.1	593.75	20.7–9264	96.8	9–100	97.1	10.1–99.9	0.6691
Grade 1	68.2	9–459.7	70.8	15–414.3	20.5	0.5–93.9	23.1	1.3–93.8	0.9087
Grade 2	420.1	14–7459.1	289.3	20.7–9264	88.5	8.4–100	88.9	10.1–99.9	0.0114
Grade 3	535.1	11.2–5109.8	569.8	20.4–8160	96.7	1–100	96.4	2.98–99.9	0.8963
Benign diseases (all)	22.5	3.2–502.7	46.7	17.8–206.5	6.7	0.7–87	8.29	1.15–85.2	0.0001
Endometriosis	45.5	6.7–377	45.2	17.8–86.7	6.6	0.7–35.34	9.1	1.2–26.16	0.2295
Teratoma tumors	15.5	6.3–51.9	46.6	26.3–724	6.48	1.65–17.9	6.76	1.87–17.6	0.2835
Follicular cysts	14.5	3.2–88	55.3	24.2–206.5	8.76	1.6–65.7	9.9	1.6–67.8	0.4886
Paraovarian cysts	13.7	4.1–52.7	48.6	36.4–186.8	7.5	3.7–87	7.9	3.8–85.2	0.7075
Hemorrhagic cysts	15.4	5.5–274.8	44.3	24.8–95.7	5.7	1.7–31.6	6.5	3.2–30.9	0.6522
AGE interval									
W1 ovarian cancer group	–	–	–	–	–	–	–	–	–
W2 ovarian cancer group	55.2	27–135.1	45.4	44.1–103.9	6.2	6–35	7.5	7.1–37.7	0.3827
W3 ovarian cancer group	403.6	98.1–1252	95.4	15–464.8	31.5	0.5–95.5	43.6	1.3–95.6	0.3619
W4 ovarian cancer group	265.4	14–4638.8	92.9	15–1500	34.7	0.5–99.7	44.7	1.3–99.4	0.7743
W5 ovarian cancer group	500	9–5887	439.3	12–1655	96.4	4.5–99.7	96.3	1.9–99.8	0.7883
W6 ovarian cancer group	233.3	9.8–7459.1	334.5	22–9264	90.9	6–100	91.6	5.2–99.9	0.9786
W7 ovarian cancer group	839	21–5659	658.4	37–8160	97	25–100	97.2	23.8–99.9	0.7710
W8 ovarian cancer group	777.5	66.9–3724	871.2	85.8–4940	97.7	55.1–100	97.9	55.1–99.9	1.0000
W1 benign group	16	4.1–274.8	41.5	24.2–84.7	6.2	1.56–22.1	6.4	1.56–22.1	0.9420
W2 benign group	28.1	7.7–377	45.5	26.3–80.4	5.4	1.65–20.3	6.2	1.87–22.7	0.0913
W3 benign group	32.3	7.1–191.5	47.3	31.7–86.7	7	2.6–24.1	9.2	3.2–26.2	0.9529
W4 benign group	21.6	5.5–168.8	44.5	17.8–74.2	6	0.7–17.6	8.9	1.15–21.3	0.0079
W5 benign group	12.4	3.2–82.1	48.5	27.5–85.1	6.9	1.8–35.3	8.4	4.4–23.4	0.6641
W6 benign group	15.9	6.7–79.8	63.8	47.1–206.5	14.3	6.7–65.7	14.1	6.6–67.8	0.6232
W7 benign group	9.55	6.3–52.7	69.5	52–186	9.7	7.3–87	9.7	7.6–85.2	1.0000

### ROC curve analysis

**Fig. 1:**
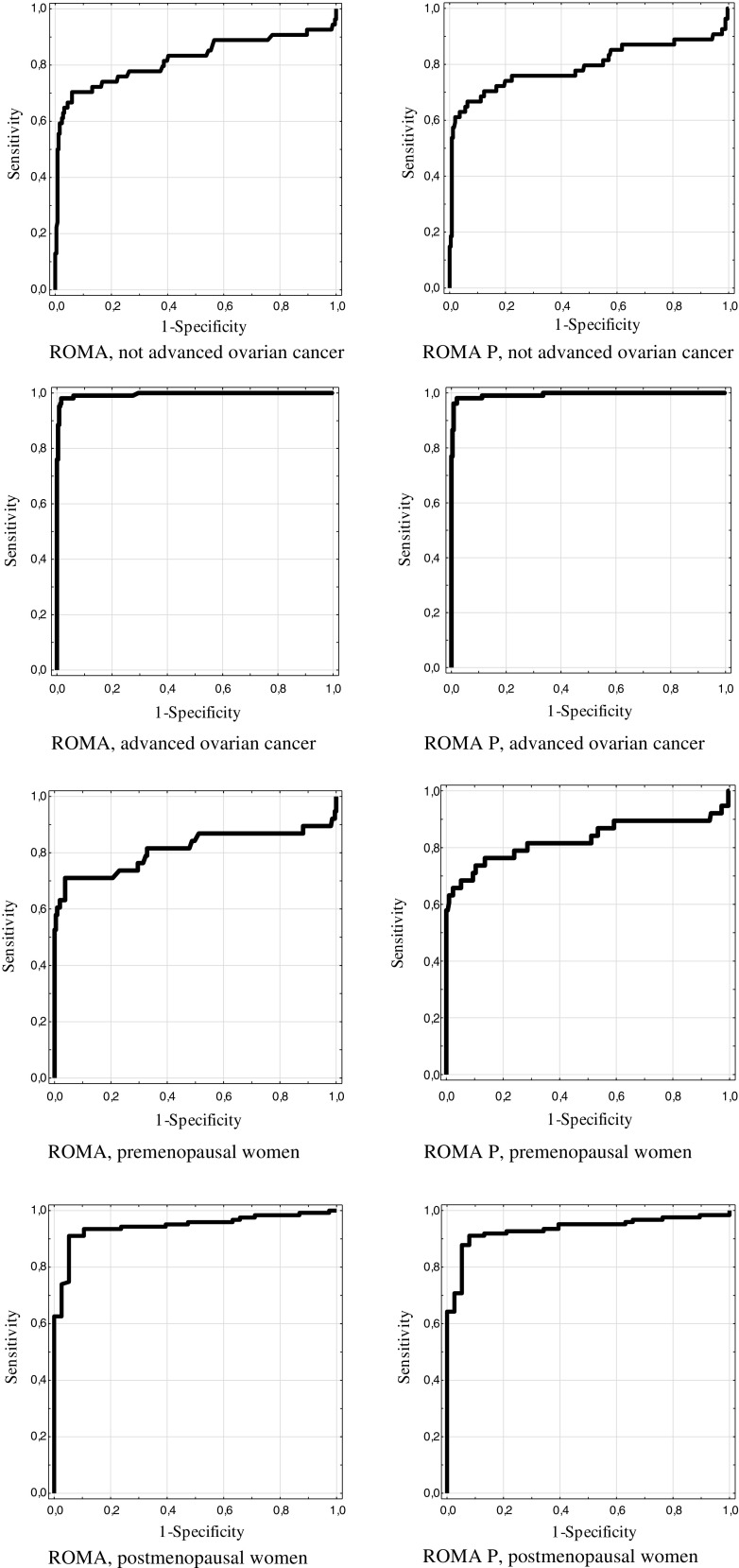
ROC curves of the standard ROMA algorithm and the ROMA P (algorithm calculated in the age ranges)

ROC curves were determined for CA125, HE4, ROMA, and ROMA P in the entire study population, in the premenopausal group, in the postmenopausal group, as well as in the groups of high- and low-stage ovarian tumors (Table 3; Fig. 1). In every case, each of the markers as well as both algorithms met the criteria of good diagnostic tests with AUC values calculated for ROC curves being above 0.5. The modified algorithm ROMA P is significantly better than CA125 in the advanced cancer group (AUC = 0.994 vs. 0.969) and better than HE4 in all cases except for the advanced cancer group. When comparing the standard ROMA algorithm and the modified ROMA P algorithm, superiority of standard algorithm was observed in the group of postmenopausal patients

**Table 3:** Values and comparisons of ROC-AUC for ROMA, ROMA P, CA125, and HE4 in studied groups

Tumor marker	ROC-AUC (95 % CI)	Comparison of ROC-AUC		
		ROMA P vs. CA125 *p* value	ROMA P vs. HE4 *p* value	ROMA P vs. ROMA *p* value
All ovarian cancer vs. benign ovarian diseases				
ROMA	0.934	0.4222	0.0004	0.1411
ROMA P	0.923			
HE4	0.881			
CA125	0.910			
Advanced ovarian cancers vs. benign ovarian diseases				
ROMA	0.995	0.0205	0.0778	0.7725
ROMA P	0.994			
HE4	0.982			
CA125	0.969			
Not-advanced ovarian cancers vs. benign ovarian diseases				
ROMA	0.820	0.7623	0.0013	0.1531
ROMA P	0.794			
HE4	0.691			
CA125	0.808			
All ovarian cancers vs. benign ovarian diseases—premenopausal patients				
ROMA	0.812	0.3393	0.0242	0.2049
ROMA P	0.829			
HE4	0.779			
CA125	0.876			
All ovarian cancers vs. benign ovarian diseases—postmenopausal patients				
ROMA	0.945	0.4277	0.0022	0.0495
ROMA P	0.935			
HE4	0.888			
CA125	0.947

ROC curves were also determined within the analyzed age ranges (Table 4). Similarly, also in this case, all analyzed parameters met the criteria of very good diagnostic tests in all age groups. There were no significant differences between the modified algorithm ROMA P and CA125, HE4, and the standard ROMA algorithm. A trend towards higher AUS values and thus towards a better diagnostic value was observed with the increasing patients’ age.

**Table 4:** Values and comparisons of ROC-AUC for ROMA, ROMA P, CA125, and HE4 in studied interval age

Age interval	Tumor marker	ROC-AUC (95 % CI)	Comparison of ROC-AUC		
			ROMA P vs. CA125 *p* value	ROMA P vs. HE4 *p* value	ROMA P vs. ROMA *p* value
W2	ROMA	0.755	0.3862	0.7901	0.6745
	ROMA P	0.745			
	HE4	0.739			
	CA125	0.673			
W3	ROMA	0.675	0.0946	0.2646	0.2545
	ROMA P	0.703			
	HE4	0.669			
	CA125	0.982			
W4	ROMA	0.863	0.9539	0.0673	0.6745
	ROMA P	0.872			
	HE4	0.826			
	CA125	0.868			
W5	ROMA	0.955	0.3405	0.0321	0.069
	ROMA P	0.926			
	HE4	0.864			
	CA125	0.956			
W6	ROMA	0.908	0.3528	0.1221	0.1468
	ROMA P	0.895			
	HE4	0.869			
	CA125	0.923			
W7	ROMA	0.96	0.3472	0.3881	1
	ROMA P	0.96			
	HE4	0.93			
	CA125	0.9			

### Sensitivity, specificity, PPV, and NPV

The cutoff value of modified algorithm ROMA P for the entire study population was calculated from receiver operating characteristic (ROC) curve and DeLong method  at the levels of 23.5 %. The cutoff value of standard algorithm ROMA was calculated based on the same statistical method at the levels of  14.1 % for the premenopausal and 25% for postmenopausal women. The sensitivity, specificity, positive predictive value and negative predictive value were calculated on the basis of contingency tables, see Table 5. The difference between the compared algorithms is evident. Marked higher sensitivity and NPV values are observed for the standard ROMA algorithm while higher specificity and PPV values are observed for the modified algorithm ROMA P.

**Table 5:** Sensitivity, specificity, PPV, and NPV of ROMA and ROMA P

Ovarian cancer vs. benign ovarian diseases	Sensitivity (%)			Specificity (%)			PPV (%)			NPV(%)		
	All	PM	M	All	PM	M	All	PM	M	All	PM	M
ROMA	88.1	75.7	91.9	84.9	92.5	89.2	87.6	63.6	96.6	92.4	95.7	76.7
ROMA P	85	64.9	91.1	98	99.1	91.9	96.5	92.3	97.4	84.8	94.2	75.6

## Discussion

In 2008, Moore et al. [12] determined that of all known biomarkers within the panel used for diagnosing ovarian cancer, HE4 was characterized by the highest sensitivity and specificity. Numerous studies demonstrated a significant improvement in the sensitivity and specificity of prediction of pathological changes within the adnexes when using CA125 and HE4 together instead of as separate markers [12, 19–22]. The sensitivity of CA125, when analyzed together with HE4, increases from 43 to 76.4 % [12]. Therefore, in 2009, Moore et al. [23] described the first predictive model for estimation of the risk of malignant epithelial ovarian cancer in women with pathological lesions within the pelvic region. In 2011, on the basis of the research of the same authors [24], the Food and Drug Administration approved the algorithm for clinical use. A continuous increase in the number of published studies on the application of ROMA in clinical practice has been observed ever since [25–29]. In 2012, during an experts’ meeting in Wiesbaden, the efficacy of ROMA was confirmed. Possibilities for improving the algorithm’s diagnostic abilities were also considered by including additional analysis of patients’ age, smoking status, renal insufficiency-related conditions, or acute cardiac insufficiency-related conditions [17].

It seems the most important factor affecting HE4 levels and thus the ROMA algorithm values is the age of the patients [3–33]. In the study by Bolstadt et al. [32], conducted in a European population, HE4 level as compared with that in women at the age of 20 was found to be increased by 2 % in women at the age of 30, 9 % in women at the age of 40, 20 % in women at the age of 50, 37 % in women at the age of 60, 63 % in women at the age of 70, and as much as 101 % in women at the age of 80. The authors suggest that HE4 levels should be analyzed in caution in patients after the age of 70. Moore et al. [31] observed the following HE4 level changes correlated with patients’ age: median level was 46.2 pmol/l before the age of 30, 43.5 pmol/L at the age of 30–39, 50.5 pmol/l after the age of 40 and before menopause, 50.7 pmol/l before the age of 60 and after menopause, 59.8 pmol/l at the age of 60–69, 66.9 pmol/l at the age of 70–79, and as much as 113.4 pmol/l after the age of 80. In our study population, HE4 levels were also found to increase with age, albeit to a smaller degree than reported by Bolstad [33] and Moore [31]. In our analysis, the increase in HE4 levels after the age of 60 as compared with the value at the age of below 20 was about 40 %. Of course, this may be due to the differences in analytical tests as Bolstad et al. [33] and Moore et al. [31] used laboratory tests from Fujirebio while our study was conducted using laboratory tests from Roche. ROMA values (modified ROMA values) were calculated using stepwise reduction of coefficients while taking into account the different age groups and differentiation of modification and directional coefficients in the ROMA algorithm calculation formula. AUC values determined for the modified algorithm ROMA P meet the criteria of a very good diagnostic test in every analysis (all ovarian cancer patients: AUC = 0.923; advanced ovarian cancer patients: AUC = 0.994; early-stage ovarian cancer patients: AUC = 0.794; premenopausal women: AUC = 0.829; and postmenopausal women: AUC = 0.935). No significant differences were observed when comparing AUC values between the standard and the modified method of algorithm calculation. However, clinically significant differences were observed when comparing specificity, PPV, and NPV. ROMA algorithm calculated according to the standard formula was characterized by better sensitivity and positive prediction values. However, the age-adjusted algorithm ROMA P proved significantly superior in terms of sensitivity and negative predictive values with only a minor reduction in sensitivity in premenopausal women only. The results in premenopausal women were as follows: sensitivity 64.9 %, specificity 99.1 %, PPV 92.3 %, and NPV 94.2. Results in postmenopausal women were 91.1, 91.9, 97.4, and 75.6 %, respectively. Compared with the results obtained by other authors who analyzed the ROMA algorithm using the standard criteria, the obtained results are very good. In their studies, Moore et al. [24] obtained the sensitivity of 88.1 %, specificity of 74.2 %, PPV of 17.8 %, and NPV of 98.3 NPV in premenopausal women compared with 90.2, 76, 56.1, and 95.8 % in postmenopausal women, respectively. Molina et al. [27] obtained analogous results in premenopausal women: 74.1, 88.9, 44.4, and 96.6 % as well as in postmenopausal women: 95.2, 83.1, 88.9, and 92.5 %.

To date, the only paper that included stratification of patients on the basis of HE4, CA125, and patient age was published several months ago [34]. It relates to a large, multicenter study in which 2665 at 8 centers were analyzed on the basis of the Copenhagen Index (CPH-I). Assuming that the cutoff value for CPH-I is at the level of 0.07, sensitivity and specificity values of 95 and 78.4 % were obtained, respectively.

## Conclusion

It appears that further studies on the improvement of diagnostic criteria of CA125- and HE4-based algorithm consisting in elimination of the impact of factors potentially affecting the values of both markers and thus of the algorithm itself, is a legitimate trend. As shown by the results of our analysis, the proposed age-related modification of algorithm calculation does not require the patients being dichotomized according to their pre- or postmenopausal status and satisfactory diagnostic values may be obtained using a single cutoff point for the entire population.
